# MLHeatmap: an interactive application for transcriptomic marker-panel discovery

**DOI:** 10.1093/bioadv/vbag213

**Published:** 2026-07-30

**Authors:** Eun Young Lee, Jihye Park, Seoyeon Youn, Kyungho Choi, Sungryul Yu, Keunsoo Kang

**Affiliations:** Department of Biomedical Sciences, College of Bio-Convergence, Dankook University, Cheonan 31116, Republic of Korea; MIND-X Superintelligence Convergence Innovation Lab, Dankook University, Cheonan 31116, Republic of Korea; Department of Biomedical Sciences, College of Bio-Convergence, Dankook University, Cheonan 31116, Republic of Korea; Department of Biomedical Sciences, College of Bio-Convergence, Dankook University, Cheonan 31116, Republic of Korea; Graduate School of Public Health, Seoul National University, Seoul 08826, Republic of Korea; Department of Clinical Laboratory Science, Semyung University, Jecheon 27136, Republic of Korea; Department of Biomedical Sciences, College of Bio-Convergence, Dankook University, Cheonan 31116, Republic of Korea; MIND-X Superintelligence Convergence Innovation Lab, Dankook University, Cheonan 31116, Republic of Korea

## Abstract

**Motivation:**

Transcriptomic biomarker studies often require separate tools for normalization, classification, feature ranking, and visualization. MLHeatmap was developed to integrate these steps into a cross-platform, browser-based workflow and to support compact marker-panel discovery directly from count matrices.

**Results:**

MLHeatmap accepts a count matrix and sample-group labels and performs gene mapping, normalization, multiclass classification with nested cross-validation, feature attribution, differential expression analysis, and interactive heatmap visualization, with support for multiple classifiers and panel-selection methods in the same workflow. As a case study, we applied MLHeatmap to colorectal cancer consensus molecular subtype (CMS) classification using 511 primary tumors from The Cancer Genome Atlas (TCGA) with published Colorectal Cancer Subtyping Consortium (CRCSC) labels. Random Forest with forward selection achieved 89.8% out-of-fold accuracy and a macro-averaged area under the receiver operating characteristic curve (macro AUC) of 0.973, yielding a 13-gene compact panel with a held-out AUC of 0.960. External validation of the compact panel gave 74.8% accuracy and a macro AUC of 0.917 in GSE39582, an independent Affymetrix GPL570 microarray cohort evaluated in 500 tumors with confident CMScaller-derived CMS assignments, and 69.18% accuracy and a macro AUC of 0.909 in CMCBSN, an independent Korean RNA-seq cohort with 159 confidently labeled tumors. Across the five classifiers and four panel-selection methods evaluated, compact-panel AUCs ranged from 0.895 to 0.970, with tree-ensemble classifiers reaching the highest values.

**Availability and implementation:**

MLHeatmap is freely available at https://github.com/kangk1204/MLHeatmap and can be installed and run locally on Windows 11, macOS, and Ubuntu.

## 1 Introduction

Transcriptomic biomarker analysis often requires users to move between several scripts and packages to complete a single study. Data preparation, normalization, group assignment, model fitting, feature ranking, panel reduction, visualization, and export are commonly handled in separate steps. Although this approach is flexible for experienced analysts, it can slow iteration and make it difficult to compare models and outputs within a single reproducible workflow.

MLHeatmap was developed to reduce this fragmentation. It provides a browser-based workflow for bulk RNA-seq count matrices. Users can upload a matrix, map genes, normalize the data, define comparison groups, run machine-learning biomarker analysis, inspect SHAP-based feature-attribution outputs, perform exploratory differential-expression analysis, render heatmaps, and export tables and plots without switching between separate tools. SHAP (SHapley Additive exPlanations; Lundberg and Lee 2017) is a game-theoretic feature-attribution method that estimates each gene’s contribution to a given sample’s predicted class. The application was built for interactive biomarker analysis rather than as a replacement for fully customized command-line workflows.

Several browser- or GUI-based tools already lower the barrier to RNA-seq analysis, including RNASeqGUI (Russo and Angelini 2014), TCC-GUI (Su *et al.* 2019), and SEQUIN (Weber *et al.* 2023), and a broader survey identified 29 such interfaces (Poplawski *et al.* 2016). These tools mainly focus on differential-expression testing, quality control, and exploratory visualization. MLHeatmap addresses a different part of the workflow: supervised multiclass classification, nested cross-validation with fold-local feature selection and panel selection, SHAP-based interpretation, compact marker-panel discovery, heatmap visualization, and export of analysis outputs across multiple classifiers and panel-selection strategies. By combining these components, MLHeatmap is intended to support supervised biomarker-panel discovery within a single interactive RNA-seq workflow.

We evaluated MLHeatmap using colorectal-cancer consensus molecular subtype (CMS) classification as a biological test case. CMS labels provide a well-established multiclass structure with distinct immune, canonical, metabolic, and mesenchymal programs (Guinney *et al.* 2015). Existing approaches, including the Colorectal Cancer Subtyping Consortium (CRCSC) consensus framework and CMScaller (Eide *et al.* 2017; Guinney *et al.* 2015), provide relevant reference points for this problem. This case study was designed to assess whether MLHeatmap could recover a compact and interpretable CMS marker panel from public transcriptomic data while retaining performance in independent external cohorts.

## 2 Methods

### 2.1 Software overview

MLHeatmap is implemented in Python and JavaScript as a local browser-based web application and can be installed and run on Windows 11, macOS, and Ubuntu. The workflow is organized into sequential steps for data upload, gene mapping, normalization, group assignment, biomarker analysis, heatmap visualization, and export. The application accepts CSV/TSV, compressed CSV/TSV, and XLSX count matrices with numeric-value validation performed during upload. Analysis settings and output are retained across visualization and export panels. The biomarker module supports Random Forest (Breiman 2001), logistic regression with L1 regularization, and linear support vector machine (SVM) in the core installation. XGBoost (Chen and Guestrin 2016) and LightGBM (Ke *et al.* 2017) are available in the full installation profile. Compact panel selection is available through forward selection, a wrapper-style greedy search (Kohavi and John 1997); lasso, based on L1-regularized logistic regression (Tibshirani 1996); stability selection (Meinshausen and Buhlmann 2010); and mRMR, minimum-redundancy maximum-relevance feature selection (Peng *et al.* 2005). Classifier and panel-selection implementations use scikit-learn (Pedregosa *et al.* 2011), with exact package and function names listed in Table 1, available as supplementary data at *Bioinformatics Advances* online. To manage runtime for larger cohorts, the biomarker step reports elapsed time and estimated time remaining, supports mid-run cancellation, and allows users to save and reuse named analysis presets across datasets.

### 2.2 Training cohort and label assignment

The Cancer Genome Atlas (TCGA) COAD and READ RNA-seq STAR count matrices were obtained from the UCSC Xena GDC hub (Goldman *et al.* 2020). Because Xena RNA-seq values are distributed as log2(count + 1), values were converted back to count scale using round(2^×^ − 1), truncated at zero, and cast to integers. Sample identifiers were restricted to primary tumors using the TCGA barcode sample-type code 01. Ensembl gene identifiers were mapped to gene symbols using the GENCODE v36 annotation map; Ensembl IDs mapping to the same gene symbol were summed to obtain one row per symbol. Published CRCSC final CMS labels, as described in the consensus CMS study (Guinney *et al.* 2015), were obtained from the public Sage Bionetworks mirror. Tumors without a final CMS label (NOLBL) were excluded, leaving 511 primary tumors for analysis.

### 2.3 MLHeatmap analysis workflow

The TCGA count matrix was analyzed in MLHeatmap using human gene mapping and DESeq2-like variance-stabilizing transform (VST) normalization. This procedure estimates median-of-ratios size factors and applies a simplified VST with a single global dispersion estimate. CMS 1–CMS4 were defined as four analysis groups. The main compact-panel analysis used Random Forest classification with forward selection and nested five-fold cross-validation. In each outer fold, the candidate pool was restricted to the top 30 genes ranked by the fitted model’s native feature importance. Forward selection then searched panels of up to 15 genes within that fold-specific candidate set. Normalization was applied to the full cohort before cross-validation as an unsupervised preprocessing step. Variance filtering, model fitting, feature-importance ranking, and panel selection were performed within each training split. SHAP values (Lundberg and Lee 2017) were computed separately on held-out outer-fold samples and used for interpretation, including the displayed/exported top-gene ranking and the SHAP heatmap. SHAP values did not determine the candidate pool or forward-selection search in the default configuration. MLHeatmap also includes an optional SHAP-based candidate-ranking mode (selection_basis = “shap”); in this mode, the fold-specific candidate pool is ranked by training-split SHAP importance. Normalization scope was evaluated by comparing the default full-cohort normalization with fold-isolated normalization. For fold-isolated normalization, normalization parameters were estimated within each outer-CV training split and then applied to the corresponding held-out fold. The two analyses were compared using accuracy, macro-averaged area under the receiver operating characteristic curve (macro AUC), paired per-class AUC differences, and panel-gene overlap. Overall accuracy was computed from outer-fold predictions of the variance-prefiltered base model. One versus rest of the ROC curves and AUC values were derived from fold-specific top-N models refit within each fold using native-importance-ranked genes. Panel selection frequency was exported as a per-fold gene-membership table. Exploratory differential-expression analysis was performed as subtype-versus-rest comparisons using Wilcoxon rank-sum testing on normalized expression.

#### 2.3.1 Batch-effect sensitivity

Because TCGA samples were generated across multiple contributing centers, each sample’s Tissue Source Site (TSS) code was used as an available site-level technical covariate. Among the 511 samples, 28 distinct TSS codes were present; 15 sites had at least 10 samples, covering 465 samples. A chi-square test was used to assess the association between TSS and CMS group. Principal component analysis was performed using the 2000 most variable genes, and η^2^ was used to quantify the proportion of PC1 and PC2 score variation associated with TSS and CMS group.

### 2.4 External validation

MLHeatmap is designed for bulk RNA-seq count matrices; microarray and other pre-normalized log-scale data were therefore evaluated outside the count-based application workflow. For external validation, the TCGA-derived compact-panel classifier was transferred to each cohort after cohort-appropriate preprocessing and gene-level standardization. Cross-platform validation used GSE39582, an Affymetrix GPL570 cohort (Marisa *et al.* 2013). Probe-level expression values were collapsed to gene-level features using platform annotation, and the shared compact-panel genes were retained. Nine of the 13 compact-panel genes were represented on GPL570; *AC005833.1*, *TDGF1*, *LY6G6F-LY6G6D*, and *LY6G6D* were absent. CMS labels were derived with CMScaller (RNAseq = FALSE, default settings, seed = 42) from log2 RMA-normalized expression values, yielding confident calls for 500 of 566 tumors. Training and test matrices were *z*-scored independently at the gene level before classifier transfer. RNA-seq external validation used CMCBSN, an independent Korean colorectal cancer cohort (Lee *et al.* 2024; Zenodo doi: 10.5281/zenodo.8333650). Of 185 annotated tumors, 159 had confident CMS calls (CMS1 = 20, CMS2 = 41, CMS3 = 41, CMS4 = 57), and 12 of the 13 compact-panel genes were represented (*LY6G6F-LY6G6D* absent). CMCBSN counts were normalized with the same DESeq2-like transform used for TCGA, shared panel genes were *z*-scored within cohort, and the Random Forest classifier trained on the full TCGA panel-gene matrix was applied without refitting.

### 2.5 Classifier and panel-method screen

To compare model settings within MLHeatmap, we evaluated five classifiers and four panel-selection methods: Random Forest, logistic regression with L1 regularization, linear SVM, XGBoost, and LightGBM, each paired with forward selection, lasso, stability, and mRMR. This produced 20 classifier-panel-selection combinations.

#### 2.5.1 Comparison with an independent classifier on the same cohort

As a controlled common-dataset comparison, we applied CMScaller (Eide *et al.* 2017), an independent published CMS classifier, to the same 511-sample TCGA cohort used for MLHeatmap model development and nested cross-validation. CMScaller was run by nearest-template prediction on the transcriptome-wide RNA-seq expression matrix using RNA-seq mode, default settings, FDR < 0.05, and seed = 42. The resulting CMScaller assignments were compared with the CRCSC reference labels and with MLHeatmap out-of-fold predictions.

## 3 Results

**Figure 1:**
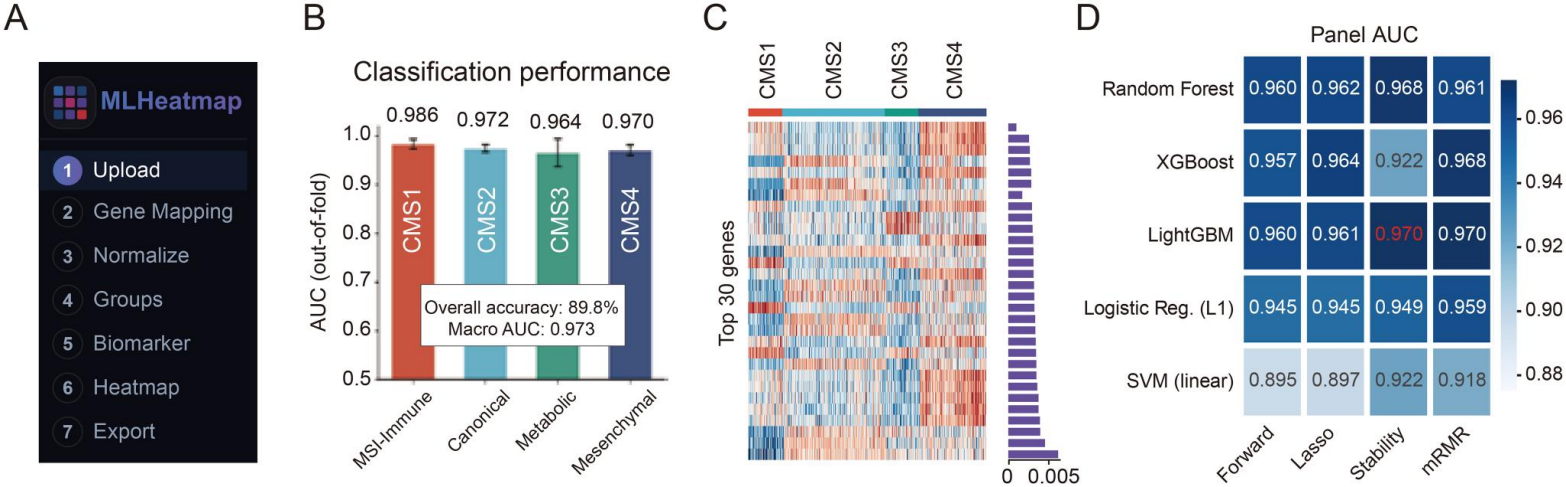
MLHeatmap workflow and CMS analysis outputs. (A) MLHeatmap user interface. (B) One versus rest AUCs for CMS classification in the TCGA cohort using Random Forest with forward selection; overall accuracy and macro AUC are shown. (C) Heatmap of marker genes with SHAP-based attribution, showing subtype-resolved expression patterns across CMS classes. SHAP values indicate each gene’s contribution to class-specific model predictions. (D) Compact-panel AUCs from the full 20-combination screen of five classifiers and four panel-selection methods.

MLHeatmap provided a single workflow from count-matrix input to compact-panel output within one interface, covering gene mapping, normalization, group assignment, biomarker analysis, differential-expression review, visualization, and export (Fig. 1A). In the CMS case study comprising 511 tumors (Fig. 1, available as supplementary data at *Bioinformatics Advances* online), Random Forest with forward selection achieved 89.8% multiclass accuracy and a macro AUC of 0.973 in the TCGA cohort, with one versus rest AUCs of 0.986 for CMS1, 0.972 for CMS2, 0.964 for CMS3, and 0.970 for CMS4 (Fig. 1B). The SHAP-ranked markers and corresponding heatmap showed subtype-resolved expression patterns across the cohort (Fig. 1C; Table 2, available as supplementary data at *Bioinformatics Advances* online). Feature ranking and panel reduction yielded a 13-gene compact panel consisting of *GAS1*, *TNNC2*, *THBS2*, *SPOCK1*, *SFRP2*, *AC005833*.1, *GALNT8*, *TDGF1*, *LY6G6F-LY6G6D*, *SFRP4*, *SLC6A4*, *LY6G6D*, and *MEOX2*, with a held-out panel AUC of 0.960 (Fig. 2, available as supplementary data at *Bioinformatics Advances* online). This panel was used as the main compact-panel example because it combined high discrimination with a small marker set and could be evaluated in independent external cohorts. In the 20-combination screen performed on the TCGA CMS cohort, compact-panel AUCs ranged from 0.895 to 0.970. Tree-ensemble classifiers reached the highest values, with LightGBM achieving an AUC of 0.970 when paired with stability selection or mRMR (Fig. 1D). This analysis illustrates how MLHeatmap can compare classifier and panel-selection settings within the same workflow.

External validation was performed with the 13-gene panel derived from the Random Forest plus forward-selection model. In GSE39582, an independent Affymetrix GPL570 cohort, 500 of 566 tumor samples received a confident CMScaller-derived CMS call. Nine of the 13 panel genes were represented on the array platform, and the transferred panel achieved 74.8% accuracy and a macro AUC of 0.917 (Table 3, available as supplementary data at *Bioinformatics Advances* online). In the independent Korean RNA-seq cohort CMCBSN, 12 of the 13 panel genes were represented, and the transferred panel achieved 69.18% accuracy and a macro AUC of 0.909 in 159 confidently CMS-labeled tumors (Table 4, available as supplementary data at *Bioinformatics Advances* online). For the common-dataset comparison, CMScaller was run on the same 511-sample TCGA cohort and agreed with the CRCSC reference labels on 88.0% of confidently assigned samples (Table 5, available as supplementary data at *Bioinformatics Advances* online). Concordance between CMScaller assignments and MLHeatmap out-of-fold predictions was 87.55% among confidently assigned samples (Table 6, available as supplementary data at *Bioinformatics Advances* online). Literature-reported CMS classifier values are shown separately as contextual reference rather than as a common-dataset benchmark (Fig. 3, available as supplementary data at *Bioinformatics Advances* online). Fold-isolated normalization gave statistically indistinguishable held-out performance compared with the default global-normalization scope (accuracy 90.4% versus 89.8%; macro AUC 0.972 versus 0.973; paired per-class AUC *P* = 0.51) (Table 7, available as supplementary data at *Bioinformatics Advances* online). The optional SHAP-based candidate-selection mode gave similar performance to the default importance-based mode (accuracy 89.8% for both; macro AUC 0.972 versus 0.973), with 12 of 13 panel genes shared between modes (Jaccard = 0.857) (Methods 2.3). A batch-effect sensitivity analysis using TCGA TSS as a site-level technical covariate found a statistically significant but modest association with CMS group (chi-square = 113.4, *df* = 42, *P* = 1.7 × 10^–8^, Cramer’s *V* = 0.285; Fig. 4, available as supplementary data at *Bioinformatics Advances* online). In PCA, TSS explained more PC1 score variation than CMS group, whereas CMS group explained more PC2 score variation than TSS. Site-associated variation was therefore detectable but did not dominate the CMS-related expression structure. Panel gene selection was consistent across the five outer cross-validation folds: *GAS1*, *TNNC2*, and *THBS2* were selected in every fold, and *SPOCK1* and *SFRP2* were selected in four of five folds (Fig. 5 and Table 2, available as supplementary data at *Bioinformatics Advances* online). Together, these analyses show that MLHeatmap can support compact marker-panel discovery, model-setting comparison, external-transfer evaluation, and robustness assessment within one analysis workflow.

## 4 Discussion

The CMS case study illustrates the use of MLHeatmap as an interactive application for transcriptomic biomarker analysis. The workflow connects count-matrix upload, gene mapping, normalization, classification, marker prioritization, heatmap visualization, differential-expression summary, and export within a single local interface. Because MLHeatmap runs locally, analyses are not dependent on hosted-server availability, queue systems, or external upload limits. Each analysis step, from normalization to classification and heatmap rendering, returns visual output in the browser, allowing users to adjust parameters and rerun without switching to a separate script.

The batch-effect sensitivity analysis identified a statistically significant but modest association between TCGA TSS and CMS group. This site-associated signal was detectable but did not dominate the CMS-related expression structure in PCA. MLHeatmap does not currently implement internal batch correction; because batch-correction parameters can introduce information leakage if estimated before cross-validation, correction for model evaluation should be estimated within the appropriate training-fold or pre-specified reference framework. The common-dataset comparison with CMScaller provided a controlled reference point beyond literature-reported cross-study values. On the identical 511-sample TCGA cohort, CMScaller agreed with the CRCSC reference labels in 88.0% of confidently assigned samples, placing MLHeatmap’s performance in the range of an independent published CMS classifier under shared-cohort evaluation.

Several practical and methodological boundaries remain. The differential-expression module is exploratory, and the DESeq2-like normalization is an approximation rather than a direct reimplementation of the DESeq2 VST (Love *et al.* 2014). MLHeatmap includes run-time controls, including elapsed time, estimated remaining time, mid-run cancellation, and reusable analysis presets, to improve usability for larger biomarker runs. Future extensions could include leakage-aware batch-correction workflows, additional external-validation utilities, and broader support for pre-normalized non-count expression data.

Within these limitations, MLHeatmap provides an integrated interactive workflow for transcriptomic biomarker analysis. In the CMS case study, the software recovered a compact and interpretable multiclass marker panel and allowed its transfer to independent external cohorts, although external-cohort accuracy was lower than internal cross-validated performance. These results support MLHeatmap as a practical tool for transcriptomic biomarker discovery while underscoring the need for cohort-specific validation.

## Supplementary Material

vbag213_Supplementary_Data
