# Supplementary material for: MLHeatmap: an interactive application for transcriptomic marker-panel discovery
Source: Bioinform Adv. 2026 Jul 30;6(1):vbag213. doi: 10.1093/bioadv/vbag213 (PMC13472729; doi:10.1093/bioadv/vbag213)
Supplement: Supplementary file 1 [file vbag213_supplementary_data.zip › Lee_Supplementary_R1.pdf]

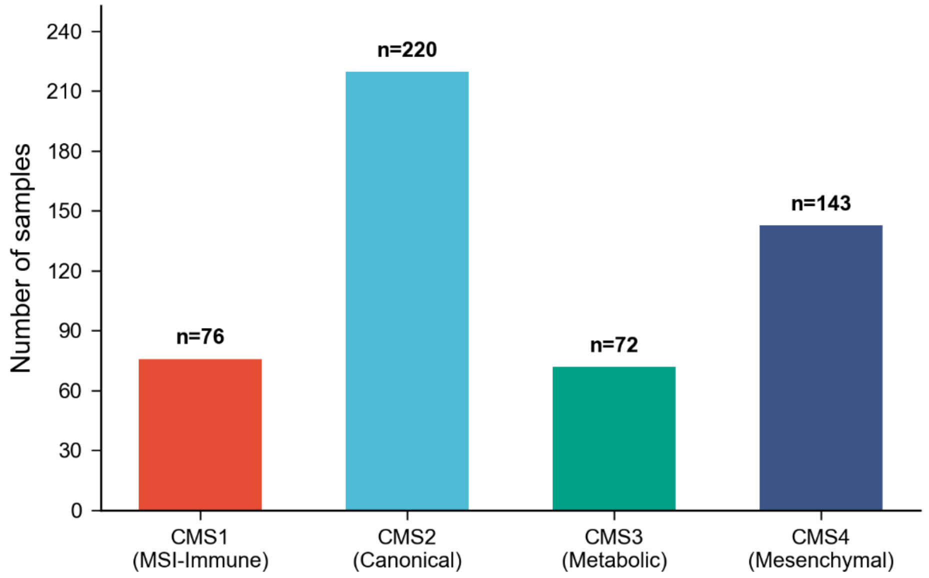

Figure S1. Distribution of consensus molecular subtypes in the TCGA colorectal cancer cohort. Bar plot showing the distribution of consensus molecular subtypes (CMS) in the TCGA colorectal cancer cohort used in this study. Samples were assigned using published CRCSC final CMS labels; samples without a final CMS assignment were excluded. The final cohort comprised 511 primary tumors, including 76 CMS1 (MSI-Immune), 220 CMS2 (Canonical), 72 CMS3 (Metabolic), and 143 CMS4 (Mesenchymal) samples.

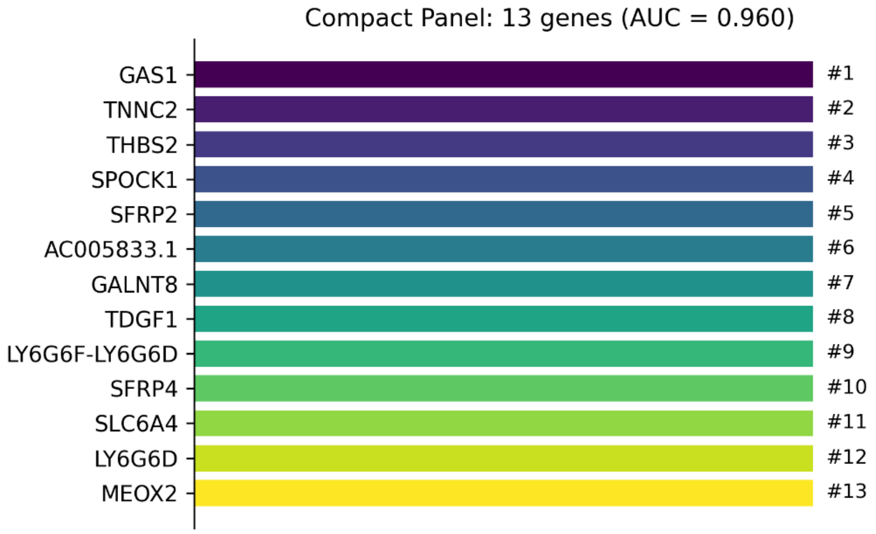

Figure S2. Compact 13-gene panel recovered by MLHeatmap. The horizontal bar plot shows the final compact panel selected using Random Forest with forward selection, ordered from #1 to #13: *GAS1*, *TNNC2*, *THBS2*, *SPOCK1*, *SFRP2*, *AC005833.1*, *GALNT8*, *TDGF1*, *LY6G6F-LY6G6D*, *SFRP4*, *SLC6A4*, *LY6G6D*, and *MEOX2*. Bar colors indicate panel rank and are used only to distinguish genes visually. The nested cross-validated panel AUC in the TCGA cohort was 0.960.

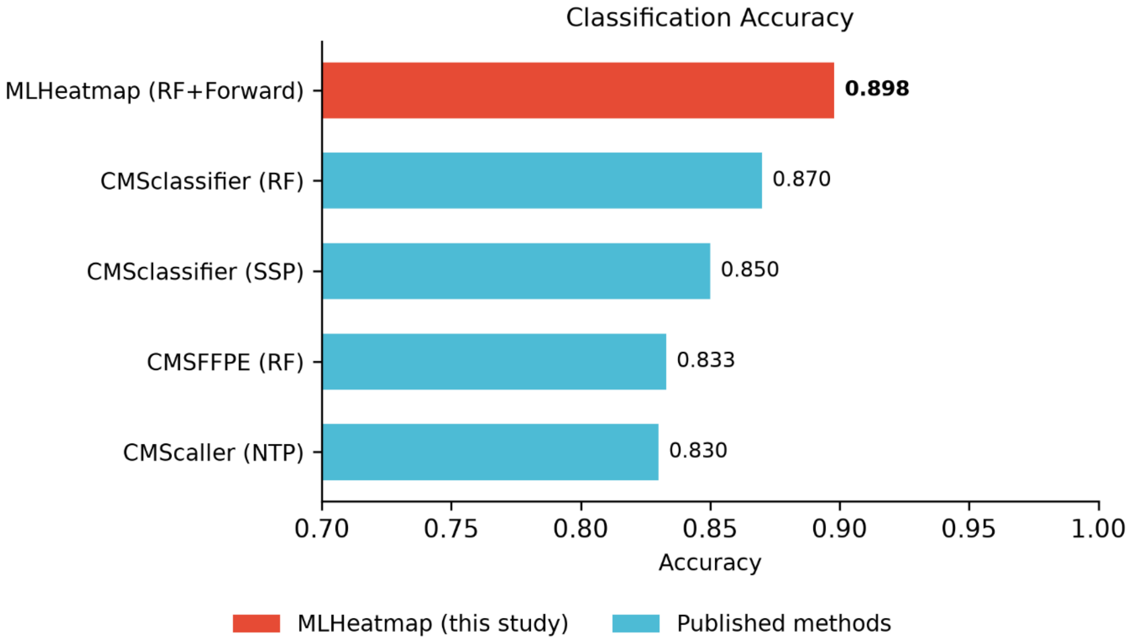

Figure S3. Literature-reported CMS classifier accuracies shown alongside the MLHeatmap TCGA out-of-fold accuracy. The MLHeatmap value is the Random Forest with forward-selection out-of-fold accuracy in the 511-sample TCGA CMS cohort. Comparator values were obtained from the original CMSclassifier, CMScaller, and CMSFFPE reports: CMSclassifier (RF), 0.870; CMSclassifier (SSP), 0.850; CMSFFPE (RF), 0.833; and CMScaller (NTP), 0.830 (Guinney et al. 2015; Eide et al. 2017; de Back et al. 2024). Because the comparator values were obtained from different cohorts, platforms, preprocessing procedures, and evaluation schemes, the figure should be interpreted as a literature context rather than as a common-dataset benchmark.

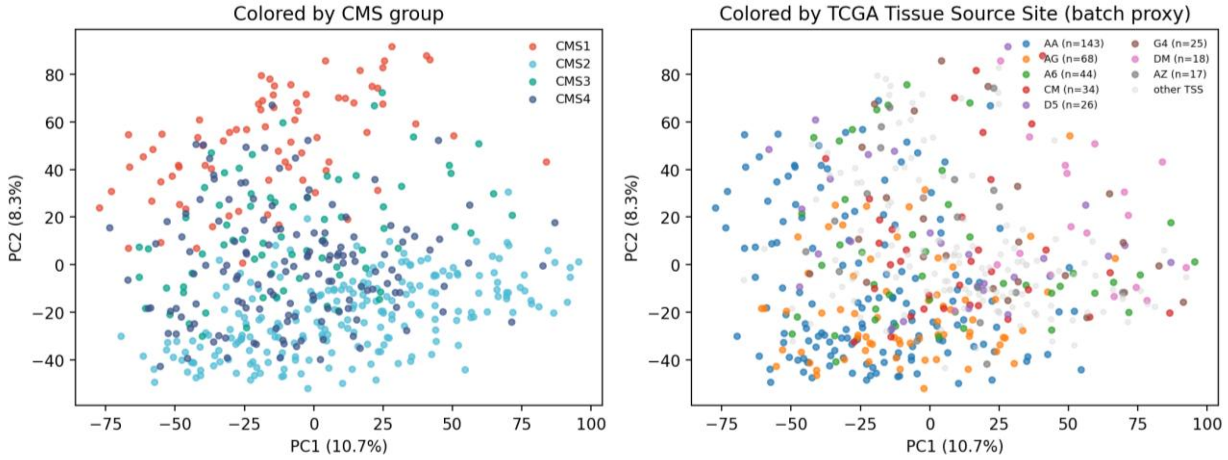

Figure S4. Batch-effect sensitivity analysis in the TCGA CRC-CMS cohort. Principal component analysis was performed using the 2,000 most variable genes after normalization. Samples are colored by CMS group in panel A and by TCGA Tissue Source Site (TSS) in panel B; the eight largest TSS

groups are shown separately, and the remaining sites are grouped as “other TSS”. TSS was significantly but modestly associated with CMS group ($\chi^2 = 113.4$, df = 42, $p = 1.7 \times 10^{-8}$, Cramer’s V = 0.285). TSS explained more PC1 variance than CMS group ($\eta^2 = 0.269$ vs. 0.094), whereas CMS group explained more PC2 variance than TSS ($\eta^2 = 0.594$ vs. 0.189). These results indicate a detectable but non-dominant site-associated signal in the cohort.

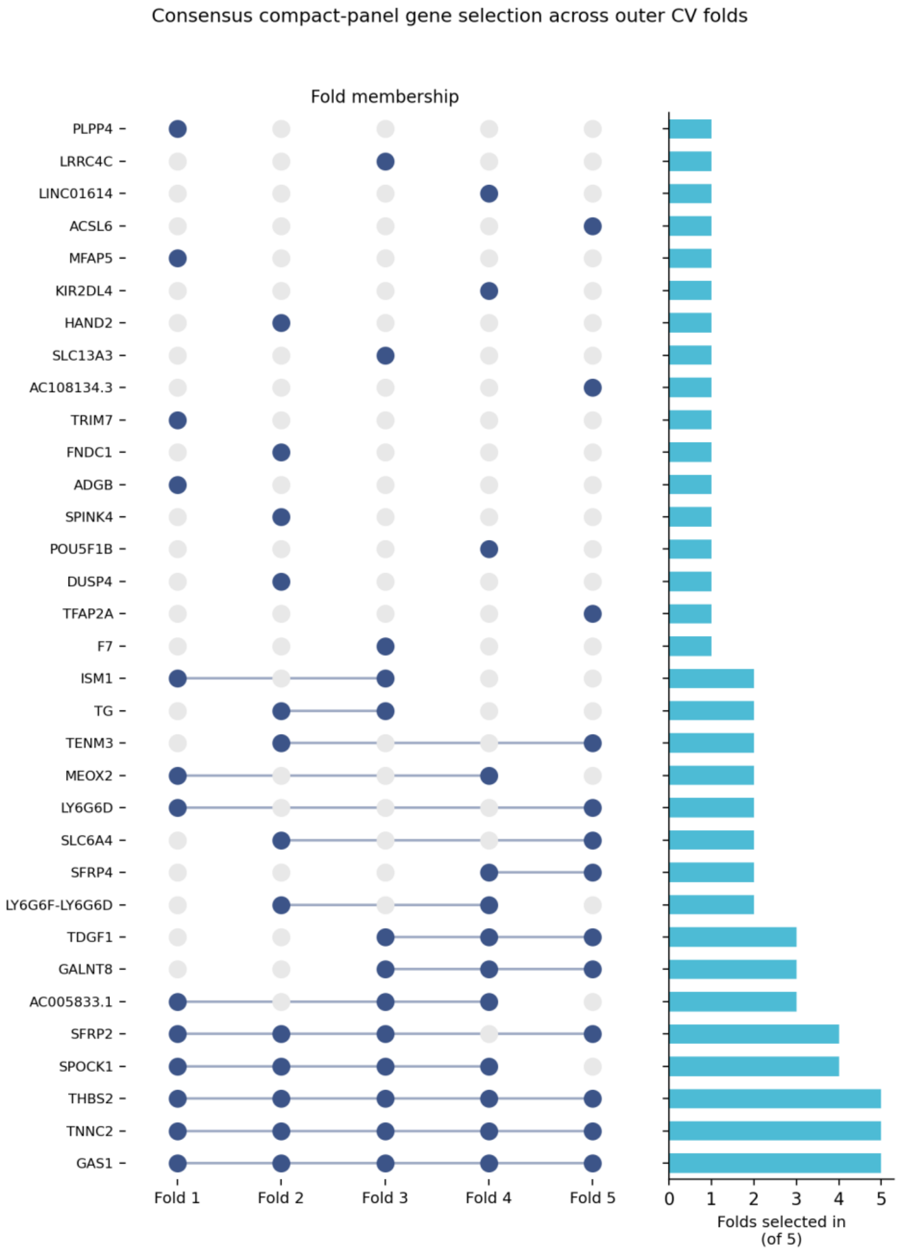

Figure S5. Cross-fold recurrence of compact-panel gene selection. The UpSet-style dot-matrix plot summarizes the 33 genes selected in at least one outer cross-validation fold. Columns indicate the five outer folds, filled and connected dots indicate selection of a gene in the corresponding fold, and the right-hand bar plot shows the number of folds in which each gene was selected. Genes are ordered by selection frequency. *GAS1*, *TNNC2*, and *THBS2* were selected in all five folds, whereas *SPOCK1* and *SFRP2* were selected in four of five folds. The underlying fold-membership data are provided in the “Panel Fold Overlap” sheet of Table S2.

Table S1. Software implementations used for classifiers, panel-selection methods, feature attribution, and normalization.

Method	Python package	Class / function
Random Forest (Breiman 2001)	scikit-learn	sklearn.ensemble.RandomForestClassifier
Logistic Regression (L1)	scikit-learn	sklearn.linear_model.LogisticRegression(penalty = 'l1', solver = 'saga')
Linear SVM	scikit-learn	sklearn.svm.SVC(kernel = 'linear')
XGBoost (Chen and Guestrin 2016)	xgboost	xgboost.XGBClassifier
LightGBM (Ke et al. 2017)	lightgbm	lightgbm.LGBMClassifier
Forward selection (Kohavi and John 1997)	scikit-learn; MLHeatmap	core/biomarker.py: _forward_order
Lasso (Tibshirani 1996)	scikit-learn; MLHeatmap	sklearn.linear_model.LogisticRegression(penalty='l1'); core/biomarker.py: _lasso_order
Stability selection (Meinshausen and Buhlmann 2010)	scikit-learn; MLHeatmap	sklearn.model_selection.StratifiedShuffleSplit + LogisticRegression; core/biomarker.py: _stability_order
mRMR (Peng et al. 2005)	scikit-learn; MLHeatmap	sklearn.feature_selection.mutual_info_classif / mutual_info_regression; core/biomarker.py: _mrmr_order
SHAP (Lundberg and Lee 2017)	shap	shap.TreeExplainer / LinearExplainer / KernelExplainer
DESeq2-like normalization	numpy/scipy; MLHeatmap	core/normalization.py: deseq2_normalize

The table lists the implementation used in the MLHeatmap analysis pipeline for each classifier, panel-selection method, attribution method, and normalization step. MLHeatmap-specific entries refer to functions in the released source code.

Table S2. MLHeatmap analysis export for the main 511-sample TCGA CMS cohort (provided as an XLSX workbook).

Table S3. External validation performance in GSE39582.

Metric	Value
Cohort	GSE39582 (Marisa et al. 2013; Affymetrix GPL570)
CMS label source	CMScaller-derived CMS assignments
Tumors with a confident CMS assignment	500 / 566
Panel genes represented / total	9 / 13 (AC005833.1, TDGF1, LY6G6F-LY6G6D, and LY6G6D were not represented on GPL570)
Accuracy	74.8%
Macro AUC	0.917

**The TCGA-derived 13-gene compact panel was evaluated in GSE39582, an independent Affymetrix GPL570 microarray cohort. CMS assignments for this cohort were re-derived. Nine of the 13 panel genes were represented on the GPL570 platform, and performance was evaluated in the 500 tumors with confident CMScaller assignments.*

Table S4. External validation performance in CMCBSN, an independent Korean RNA-seq cohort.

Metric	Value
Cohort	CMCBSN (Lee et al. 2024, Mol Cells; Zenodo doi:10.5281/zenodo.8333650)
Platform	RNA-seq (RSEM expected counts)
Annotated tumors	185
Samples with confident CMS assignments	159 (CMS1=20, CMS2=41, CMS3=41, CMS4=57)
Panel genes represented / total	12 / 13 (*LY6G6F-LY6G6D* was not represented)
Accuracy	69.18%
Macro AUC	0.909

The TCGA-derived 13-gene panel was transferred to CMCBSN using the same normalize-then-z-score-then-classify procedure applied to GSE39582 (Methods 2.4). Accuracy was lower in CMCBSN than in GSE39582 (69.18% vs. 74.8%), whereas macro AUC was similar across the two external cohorts (0.909 vs. 0.917). This pattern suggests reduced calibration across cohorts while preserving subtype-discriminative ranking performance. The published CMCBSN CMS annotations were used as the reference endpoint; as a label-provenance check, rerunning CMScaller on the CMCBSN tumor expression matrix reproduced the published CMS1–CMS4 assignments exactly (159/159), with the same 26 tumors left unassigned.

Table S5. Common-dataset comparison of CMScaller against CRCSC reference labels in the 511-sample TCGA cohort.

Metric	Value
Samples analyzed	511
Confident CMScaller calls	466/511 (91.2%)
Agreement with CRCSC reference labels	410/466 (88.0%) among confident assignments
Agreement with CRCSC reference labels	410/511 (80.2%) when non-confident assignments were counted as incorrect

CMScaller (Eide et al. 2017) was run on the same 511-sample TCGA count matrix used for MLHeatmap model development and evaluation, using nearest-template prediction with RNAseq = TRUE, default settings, FDR < 0.05, and seed = 42. Among confident CMScaller assignments, the confusion matrix against CRCSC reference labels was: CMS1, 69/0/3/2; CMS2, 1/151/9/25; CMS3, 8/0/55/0; and CMS4, 1/3/4/135. Rows indicate CRCSC reference labels, and columns indicate CMScaller assignments. The largest off-diagonal component was between CMS2 and CMS4, consistent with the known difficulty of this subtype boundary. The corresponding comparison with MLHeatmap out-of-fold predictions is shown in Table S6.

Table S6. Concordance between MLHeatmap out-of-fold predictions and CMScaller assignments in the 511-sample TCGA cohort.

Metric	Value
Samples analyzed	511
MLHeatmap out-of-fold accuracy against CRCSC reference labels	89.82%
CMScaller accuracy against CRCSC reference labels among confident assignments	88.0% (466/511 confident assignments)
MLHeatmap–CMScaller agreement among confident CMScaller assignments	87.55%

MLHeatmap and CMScaller showed broadly concordant subtype assignments on the same TCGA cohort. Agreement between the two classifiers among samples with confident CMScaller assignments was similar to each classifier’s agreement with the CRCSC reference labels.

Table S7. Normalization-scope sensitivity analysis comparing global and fold-isolated normalization in the 511-sample TCGA cohort.

Metric	Global normalization	Fold-isolated normalization
Accuracy (out-of-fold, TCGA)	89.8%	90.4%
Macro AUC (out-of-fold, TCGA)	0.973	0.972
Compact panel size	13 genes	12 genes
Panel AUC (held-out)	0.960	0.961
Paired per-class AUC difference	—	t=0.74, p=0.51; n = 4 CMS classes
Panel gene Jaccard overlap with global scope	—	0.47

Global and fold-isolated normalization produced similar held-out performance in the TCGA cohort. The paired per-class AUC comparison across the four CMS classes showed no significant difference between normalization scopes (t = 0.74, p = 0.51). The compact panels differed more in gene composition than in performance, with a Jaccard overlap of 0.47 between the global and fold-isolated panels.

References

Breiman L. Random forests. Mach Learn 2001;45(1):5–32. https://doi.org/10.1023/A:1010933404324.

Chen T, Guestrin C. XGBoost: a scalable tree boosting system. In: Proceedings of the 22nd ACM SIGKDD International Conference on Knowledge Discovery and Data Mining. 2016:785–94. https://doi.org/10.1145/2939672.2939785.

de Back TR, Wu T, Schafrat PJM et al. A consensus molecular subtypes classification strategy for clinical colorectal cancer tissues. Life Sci Alliance 2024;7(8):e202402730. https://doi.org/10.26508/lsa.202402730.

Eide PW, Bruun J, Lothe RA, Sveen A. CMScaller: an R package for consensus molecular subtyping of colorectal cancer pre-clinical models. Sci Rep 2017;7(1):16618. https://doi.org/10.1038/s41598-017-16747-x.

Guinney J, Dienstmann R, Wang X et al. The consensus molecular subtypes of colorectal cancer. Nat Med 2015;21(11):1350–6. https://doi.org/10.1038/nm.3967.

Harris CR, Millman KJ, van der Walt SJ et al. Array programming with NumPy. Nature 2020;585(7825):357–62. https://doi.org/10.1038/s41586-020-2649-2.

Ke G, Meng Q, Finley T et al. LightGBM: a highly efficient gradient boosting decision tree. Adv Neural Inf Process Syst 2017;30.

Kohavi R, John GH. Wrappers for feature subset selection. Artif Intell 1997;97(1–2):273–324. https://doi.org/10.1016/S0004-3702(97)00043-X.

Lee J, Kim JH, Chu HBK et al. Comprehensive RNA-sequencing analysis of colorectal cancer in a Korean cohort. Mol Cells 2024;47(3):100033. https://doi.org/10.1016/j.mocell.2024.100033.

Love MI, Huber W, Anders S. Moderated estimation of fold change and dispersion for RNA-seq data with DESeq2. Genome Biol 2014;15(12):550. https://doi.org/10.1186/s13059-014-0550-8.

Lundberg SM, Lee SI. A unified approach to interpreting model predictions. Adv Neural Inf Process Syst 2017;30.

Marisa L, de Reyniès A, Duval A et al. Gene expression classification of colon cancer into molecular subtypes: characterization, validation, and prognostic value. PLoS Med 2013;10(5):e1001453. https://doi.org/10.1371/journal.pmed.1001453.

Meinshausen N, Bühlmann P. Stability selection. J R Stat Soc Series B Stat Methodol 2010;72(4):417–73. https://doi.org/10.1111/j.1467-9868.2010.00740.x.

Pedregosa F, Varoquaux G, Gramfort A et al. Scikit-learn: Machine Learning in Python. J Mach Learn Res 2011;12:2825–30.

Peng H, Long F, Ding C. Feature selection based on mutual information criteria of max-dependency, max-relevance, and min-redundancy. IEEE Trans Pattern Anal Mach Intell 2005;27(8):1226–38. https://doi.org/10.1109/TPAMI.2005.159.

Tibshirani R. Regression shrinkage and selection via the lasso. J R Stat Soc Series B Methodol 1996;58(1):267–88. https://doi.org/10.1111/j.2517-6161.1996.tb02080.x.

Virtanen P, Gommers R, Oliphant TE et al. SciPy 1.0: fundamental algorithms for scientific computing in Python. Nat Methods 2020;17(3):261–72. https://doi.org/10.1038/s41592-019-0686-2.
